# Nucleolin, a Shuttle Protein Promoting Infection of Human Monocytes by *Francisella tularensis*

**DOI:** 10.1371/journal.pone.0014193

**Published:** 2010-12-01

**Authors:** Monique Barel, Karin Meibom, Alain Charbit

**Affiliations:** 1 Université Paris Descartes, Faculté de Médecine Necker Enfants-Malades, Paris, France; 2 INSERM U1002, Unit of Pathogenesis of Systemic Infections, Paris, France; East Carolina University School of Medicine, United States of America

## Abstract

**Background:**

*Francisella tularensis* is a highly virulent facultative intracellular bacterium, disseminating in vivo mainly within host mononuclear phagocytes. After entry into macrophages, *F. tularensis* initially resides in a phagosomal compartment, whose maturation is then arrested. Bacteria escape rapidly into the cytoplasm, where they replicate freely. We recently demonstrated that nucleolin, an eukaryotic protein able to traffic from the nucleus to the cell surface, acted as a surface receptor for *F. tularensis* LVS on human monocyte-like THP-1 cells.

**Methodology/Principal Findings:**

Here, we followed the fate of nucleolin once *F. tularensis* has been endocytosed. We first confirmed by siRNA silencing experiments that expression of nucleolin protein was essential for binding of LVS on human macrophage-type THP-1 cells. We then showed that nucleolin co-localized with intracellular bacteria in the phagosomal compartment. Strikingly, in that compartment, nucleolin also co-localized with LAMP-1, a late endosomal marker. Co-immunoprecipation assays further demonstrated an interaction of nucleolin with LAMP-1. Co-localization of nucleolin with LVS was no longer detectable at 24 h when bacteria were multiplying in the cytoplasm. In contrast, with an *iglC* mutant of LVS, which remains trapped into the phagosomal compartment, or with inert particles, nucleolin/bacteria co-localization remained almost constant.

**Conclusions/Significance:**

We herein confirm the importance of nucleolin expression for LVS binding and its specificity as nucleolin is not involved in binding of another intracellular pathogen as *L. monocytogenes* or an inert particle. Association of nucleolin with *F. tularensis* during infection continues intracellularly after endocytosis of the bacteria. The present work therefore unravels for the first time the presence of nucleolin in the phagosomal compartment of macrophages.

## Introduction

*Francisella tularensis* is a small non-motile Gram-negative bacterium that causes the zoonotic disease tularemia in a large number of animals, such as rabbits, hares, and small rodents [1]. *F. tularensis* is also one of the most infectious human bacterial pathogens as ten bacteria can cause disease in humans [1], [2]. Humans acquire infection by direct contact with sick animals, inhalation, ingestion of contaminated water or food, or by bites from ticks, mosquitoes or flies. *F. tularensis* has significant potential as an agent of bioterrorism due to its infectivity and capacity to infect in form of aerosols and its ability to cause illness and death [1].

Three subspecies (subsp) are pathogenic for humans: *F. tularensis* subsp *tularensis* (type A strain), *F. tularensis* subsp *holarctica* (type B strain) and *F. tularensis* subsp *mediasiatica*. They differ in terms of their pathogenicity and geographic origin, but are phylogenetically very closely related. The fourth subsp, *novicida* provokes disease in mice, but is rarely pathogenic in humans. *F. tularensis* live vaccine strain (LVS) is an attenuated type B strain [3]. *F. tularensis* is a highly virulent facultative intracellular bacterium, disseminating within host mononuclear phagocytes. After entry into macrophages, *F. tularensis* initially resides in a phagosomal compartment, whose maturation is then arrested. Bacterial escape into the cytoplasm, initially reported to occur after 2–6 hours of infection, has now been observed as early as 30–60 min after phagocytosis [4]. Bacteria then replicate freely in the cytoplasm of the macrophages [3], [5]. Bacteria are ultimately released from infected cells after induction of apoptosis and pyropoptosis [6]–[8].

Among the mechanisms that mediate uptake of *F. tularensis* by phagocytic cells, participation of C3 [9], CR3 [10], class A scavenger receptors [11] and mannose receptor [12], have been reported. More recently, we have shown that nucleolin, an eukaryotic protein able to traffic from the nucleus to the cell surface acted as a surface receptor for *F. tularensis* LVS on human monocyte-like THP-1 cells [13]. We also demonstrated that the ligand for human nucleolin at the bacterial surface was the elongation factor Tu (EF-Tu) and that EF-Tu interacted specifically with the C-terminal RGG domain of nucleolin.

In the present work, we were interested in the fate of nucleolin after *F. tularensis* LVS entry in cells. We first confirmed by siRNA silencing experiments that expression of nucleolin was essential for binding and infection by LVS of human monocyte/macrophage-type cells. Down-regulation of nucleolin expression had no effect on binding of *Listeria monocytogenes* or inert particles to human cells. We then tracked nucleolin localization at different time points of infection, by confocal microscopy analysis. We found that nucleolin co-localized with intracellular bacteria at a high level in the phagosomal compartment. This co-localization strongly decreased when the bacteria reached the cytosol to multiply.

## Results and Discussion

### Down-regulation of nucleolin expression decreases LVS binding and infection

**Figure 1:**
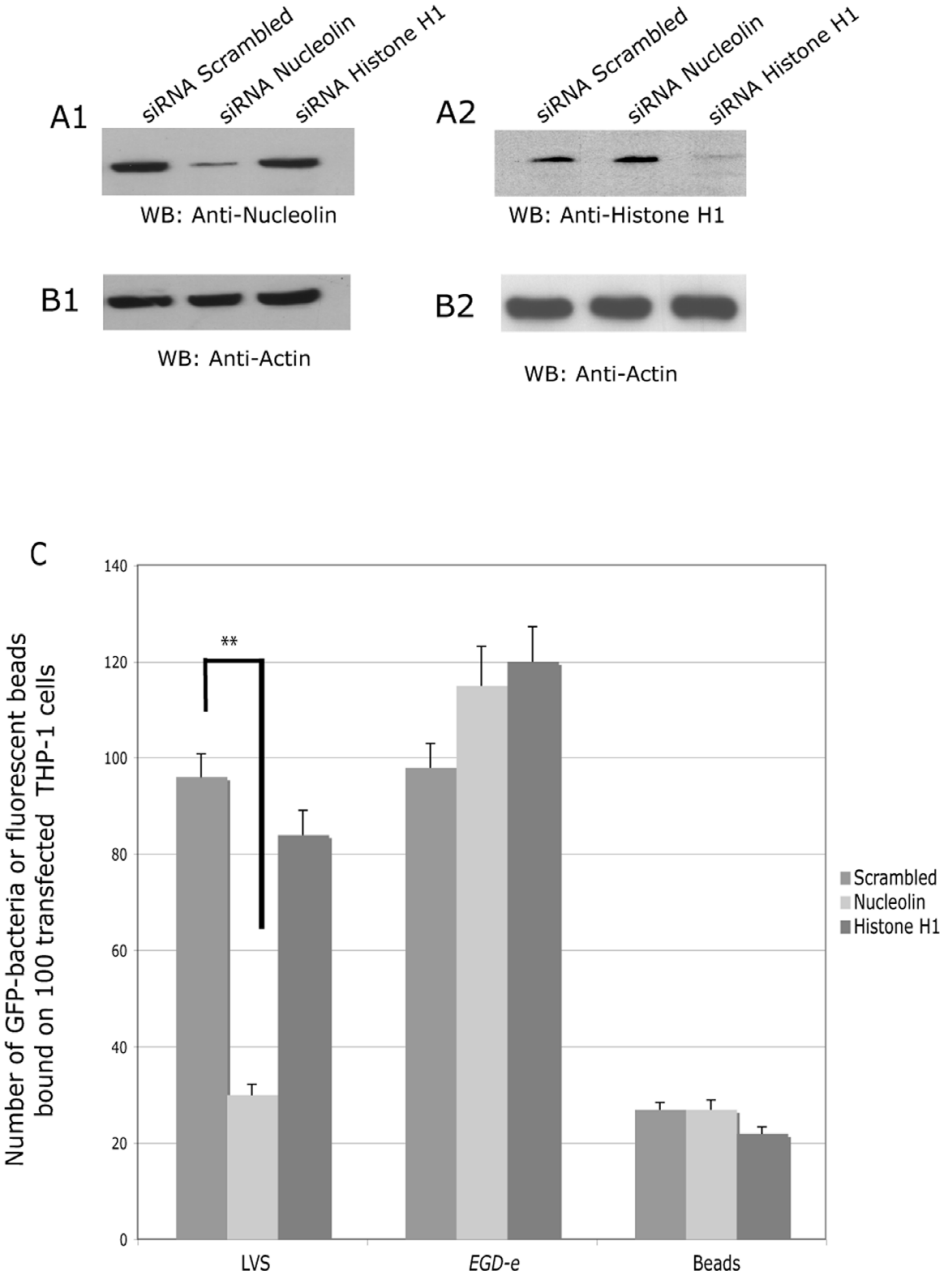
Down-regulation of nucleolin expression decreases binding to human monocytes. THP-1 cells were transfected with siRNA either specific of nucleolin (siRNA-nucleolin), histone H1 (siRNA-Histone H1) or non-specific (siRNA-scrambled) for 72 h. Proteins were solubilized from pelleted transfected cells and analyzed by immunoblotting technics with anti-nucleolin MoAb (**part A1**), anti-Histone H1 (**part A2**) or anti-actin Ab (**parts B1 and B2**). Quantification of LVS-GFP, *L. monocytogenes* (EGD-e) or fluorescent beads binding on transfected cells (**part C**) was performed after confocal analysis of cells fixed with PFA and mounted in Mowiol. Results show the mean from three independent experiments, ± SD values indicated as error bars. ** p value <0.001 (two-tailed unpaired Student's *t* test).

We have previously shown [13] that nucleolin, expressed on human cell surface, was involved in LVS infection. To confirm that expression of nucleolin was essential for LVS binding on human cells, we performed silencing RNA experiments, using siRNA specifically knocking down nucleolin (**Fig. 1**). We controlled the specificity of the assay: i) by using a siRNA knocking down another eukaryotic protein, histone H1, which has been associated with many effects of nucleolin [14], [15]; and ii) by monitoring entry of either another intracellular pathogen with a cytosolic niche, *Listeria monocytogenes*, or of fluorescent beads, with the same average 0.5 µm diameter as *F*. *tularensis*. Efficiency of THP-1 cell infection by *L. monocytogenes* was confirmed by confocal microscopy and by CFU counting (data not shown). Expression of either nucleolin or histone H1 (**Fig. 1A1 and 1A2**) was significantly decreased in cells transfected with siRNA-Nucleolin or siRNA-histone H1, respectively. Expression of nucleolin was not affected in cells transfected with siRNA-histone H1 or non-specific siRNA-scrambled. Likewise, expression of histone H1 was not affected in cells transfected with siRNA-nucleolin or non-specific siRNA-scrambled. The expression level of actin was not changed in all these assays (**Fig. 1B1 and 1B2**). Binding of GFP-expressing bacteria or fluorescent beads to THP-1 cells that had been transfected with the different siRNAs was analyzed by confocal microscopy. Quantification was performed with Image J software (**Fig. 1C**). Binding of LVS-GFP decreased by 70% when cells were transfected with siRNA-nucleolin. Neither siRNA-scrambled nor siRNA-histone H1 had an effect on LVS-GFP binding. Binding of *L. monocytogenes*-GFP or fluorescent beads stayed at the same maximum level whether the cells were transfected with siRNA-nucleolin or siRNA-histone H1. These results demonstrated that expression of nucleolin is essential for LVS binding and that nucleolin does not participate to the binding of another pathogen like *L. monocytogenes* or inert particles like fluorescent beads.

### Intracellular localization of nucleolin after bacterial entry

As nucleolin is able to shuttle from plasma membranes to the nucleus [16], [17], we followed its localization after LVS bacteria had been endocytosed at 1 h, 5 h and 24 h, by confocal microscopy. To distinguish between the cytoplasmic and phagosomal compartments, we used the differential solubilization process previously described [18]. Briefly, the plasma membrane is selectively permeabilized with digitonin (**Fig. S1A and S1B**). This treatment allows the detection of cytoplasmic bacteria and proteins. Subsequent treatment with saponin renders intact phagosomes accessible to antibodies (**Fig. S1C**) and allows detection of intra-phagosomal bacteria.

**Figure 2:**
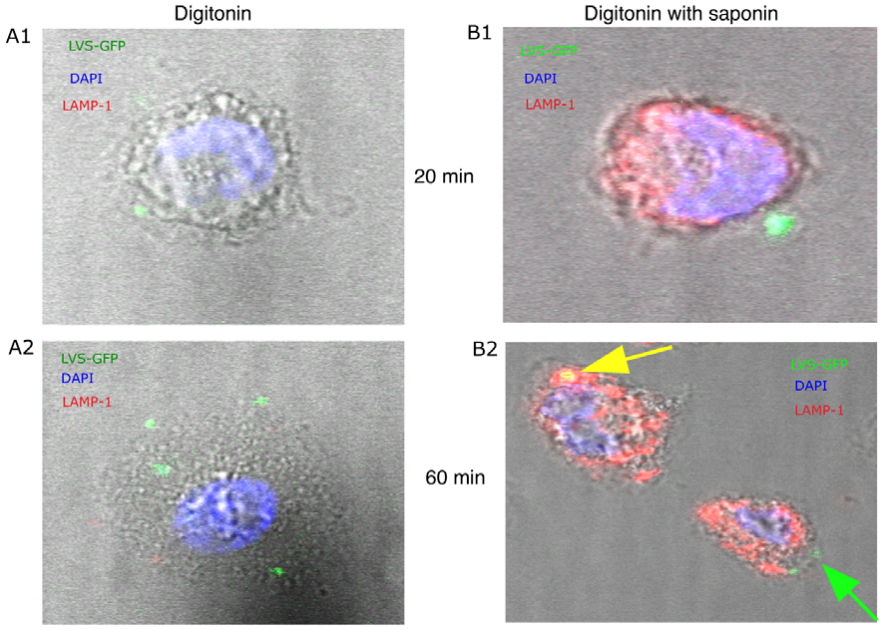
Differential treatment of cells with digitonin without or with saponin. Adherent THP-1 cells were infected by LVS-GFP (**green**) for 20 min (**A1 and B1**) or 60 min (**A2 and B2**) and permeabilized in digitonin. Cells (**A1 and A2**) were then incubated with anti-LAMP-1 Ab and Alexa 546 GAR (**red**). Cells (**A1, A2, B1 and B2**) were fixed and further incubated (**B1 and B2**) with anti-LAMP-1 and Alexa Fluor 546 GAR both in saponin. DAPI (**blue**) was added to all cells. After mounting the glass coverslips in Mowiol, cells were visualized by confocal microscopy. We present the photographs of cells with typical morphology and staining. Green arrow: LVS-GFP in cytoplasm; yellow arrow: LVS-GFP co-localized with LAMP-1.

**Figure 3:**
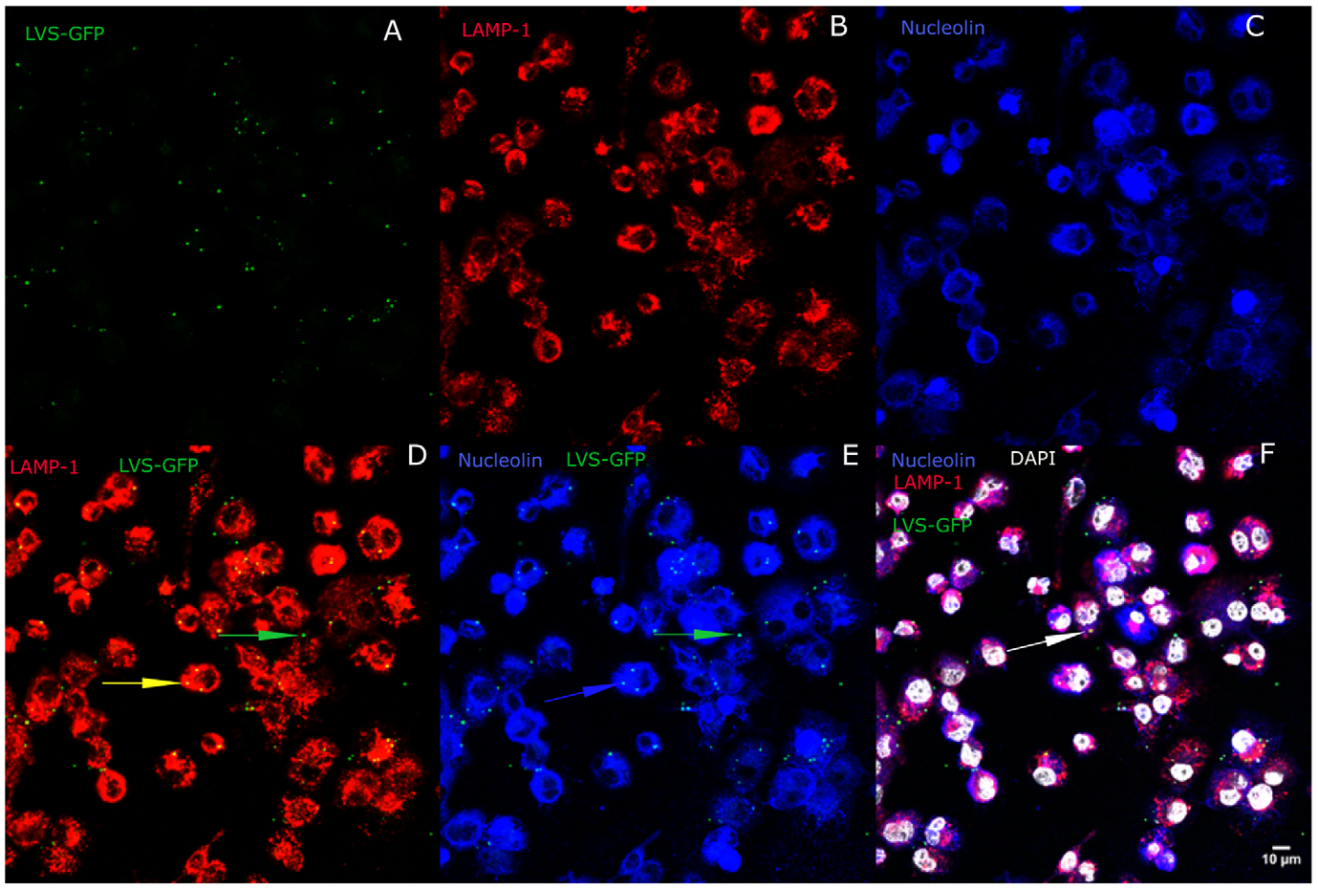
Intracellular co-localization of nucleolin with LVS and LAMP-1. Adherent THP-1 cells infected for 1 h by LVS-GFP (**Part A, green**) were permeabilized in digitonin. Cells were then fixed and incubated with anti-LAMP-1 Ab (**Part B, red**) or anti-nucleolin MoAb (**Part C, blue**) in saponin. After washing, cells were incubated with Alexa Fluor 546 GAR (**red**) and Cy5-conjugated GAM (**blue**) in saponin. DAPI (**white**) was added in all cells. Cells on glass coverslips were mounted in Mowiol and were visualized by confocal microscopy. The merging of LVS-GFP (**Part A**) either with LAMP-1 (**Part D**) or nucleolin (**Part E**) or with both LAMP-1 and nucleolin (**Part F**) was performed using the Magic Montage module of Image J software. Green arrows point to LVS-GFP bacteria in cytoplasm, yellow arrow points to LVS-GFP bacteria co-localized with LAMP-1, blue arrow points to LVS-GFP co-localized with nucleolin and white arrow points to LVS-GFP bacteria co-localizing with both nucleolin and LAMP-1. We present the photographs of cells with typical morphology and staining.

**Figure 4:**
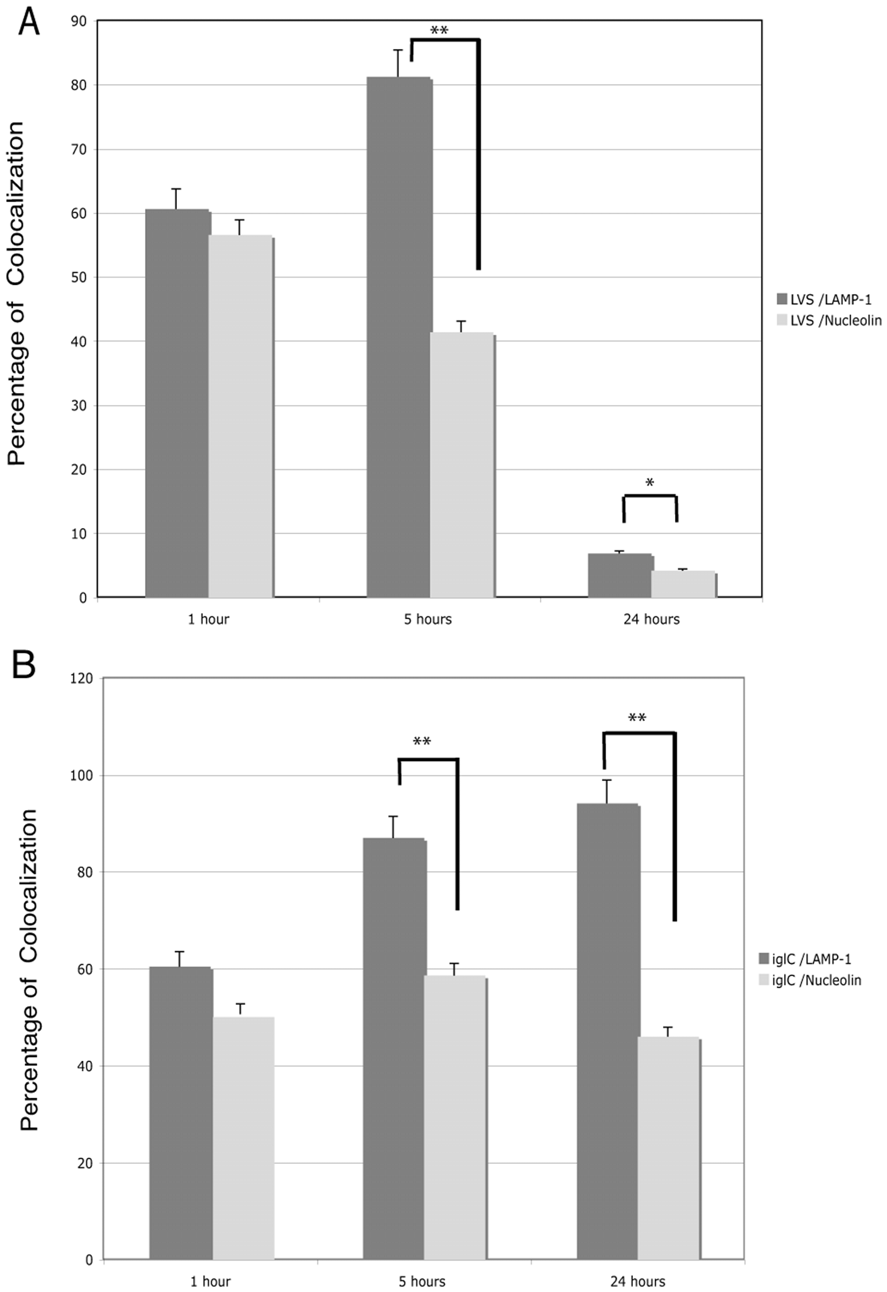
Quantification of intracellular interaction of bacteria with nucleolin or LAMP-1. Adherent THP-1 cells were infected either with LVS-GFP (**Part A**) or *iglC*-GFP (**Part B**) and further incubated for different times. Labeling was performed as described in Figure 3 and quantification of labeling was obtained with Metamorph software. These graphs result from analysis of six different fields for each time of infection, in five independent experiments. Data are expressed as the mean percentage (± SD) of bacteria co-localizing either with nucleolin or with LAMP-1. * p value <0.01, ** p value <0.001 (two-tailed unpaired Student's *t* test).

As the timing of bacteria entry into the phagosomes varies with cell type [10], [18], we looked at entry of bacteria into phagosomes during the first hour post-infection (**Fig. 2**). Whatever the time of infection, the LAMP-1 protein, a specific marker of phagosomes [19], [20], was detected only after treatment of infected cells by both digitonin and saponin (**Fig. 2B1 and 2B2**), and not when only digitonin was used (**Fig. 2A1 and 2A2**). This confirmed the efficiency of detergent treatment. At 20 min (**Fig. 2A1, 2B1**), LVS-GFP bacteria have been already internalized and are visible near the cell surface. At 1 h (**Fig. 2A2, 2B2**), LVS-GFP bacteria are found already co-localized with LAMP-1. So our further observations by confocal microscopy of LVS-GFP co-localization with the different proteins were made after 1 h or later during the infection. Intracellular localization at 1 h of LVS-GFP (**Fig. 3A**), LAMP-1 (**Fig. 3B**) or nucleolin (**Fig. 3C**) was analyzed using specific antibodies and their co-localization was monitored (**Fig. 3Dor 3E** **, respectively**). Merging of nucleolin, LAMP-1 and LVS-GFP was also obtained (**Fig. 3F**). Quantification of each co-localization was then performed with Metamorph software (**Fig. 4A**). LVS/LAMP-1 co-localization was observed already at 1 h, with a peak observed at 5 h, where 81% ±5% of LVS co-localized with LAMP-1. This co-localization decreased to 6% ±1% at 24 h. LVS/nucleolin co-localization continuously decreased, from 56% ±4% at 1 h to 4% ±1% at 24 h. These results were obtained only in presence of both digitonin and saponin, confirming that LAMP-1 could serve as a marker for LVS localization in phagosomes [18]. Furthermore, these data demonstrated that nucleolin also co-localized with *F. tularensis* LVS, after its endocytosis. The bacteria/nucleolin co-localization decreased concomitantly with infection from the beginning of the infection at 1 h until 24 h, when LVS bacteria multiply in the cytoplasm. This decrease could be due either to a decrease in expression level of nucleolin or to a dissociation of nucleolin from LVS. Notably, we observed that the level of nucleolin detected in the cells was constant up to 24 h after infection (data not shown). We therefore hypothesize that the decrease in nucleolin/LVS co-localization at 24 h may reflect the dissociation of nucleolin from LVS, after the bacteria have escaped from the phagosome. All these experiments have been performed using bacteria transformed with a plasmid expressing GFP from the *groEL* promoter. We did not test each strain for the percentage of bacteria expressing GFP, which could be a possible variable, as described in other pathogens [21]. However, this should not have affected the outcome of our experiments as the different times of infection were performed with the same batch of bacteria for each experiment.

By comparison, we looked at the fate of intracellular nucleolin in human macrophage-like cells infected with a *Francisella iglC* mutant, which has a severe defect in phagosomal escape and intracellular replication [19], [20], [22]. We first verified that the *iglC* mutant was not multiplying in THP-1 macrophage-like cells by CFU counting at 24 h (data not shown) and analysis by confocal fluorescence (**Fig. S2**). Quantification of co-localization (**Fig. 4B**) showed that 60% ±4% of *iglC-*GFP were co-localizing with LAMP-1 at 1 h. At 5 h, 85% ±5% *iglC*-GFP were co-localizing with LAMP-1, the same level as obtained after LVS infection. However, at 24 h, in opposition to the important decrease observed with LVS, 90% ±5% *iglC*-GFP were still co-localizing with LAMP-1. Co-localization of *iglC* bacteria with nucleolin remained constant from 1 h to 24 h of infection, at an average level of 50% ±8%. Thus, in LVS infection, where bacteria egress rapidly from phagosomes, co-localization of bacteria either with LAMP-1 or with nucleolin diminished with time. In contrast, with the *iglC* mutant that remains trapped in the phagosomal compartment, co-localization of the bacteria either with LAMP-1 or nucleolin remained at a constant level during the infection. Notably, the co-localization of both wild-type LVS and *iglC* mutant strains was always more important with LAMP-1 than with nucleolin, at all time points tested. It was especially more prominent at 5 h. This result could be explained by a higher amount of LAMP-1 protein present in phagosomes. Co-localization of LVS or the *iglC* mutant strain with LAMP-1 has been already described [3], [18], [20]. We found here that LVS and the *iglC* mutant co-localized with LAMP-1 at a higher level (85%) at 5 h than at 1 h (60%). This timing slightly differs from that observed by others [3] and is likely due to different infection conditions, as e.g. our time 1 h corresponds to their time zero.

These results support the notion that *F. tularensis* co-localization with nucleolin after its endocytosis is related to the capacity of the bacteria to escape from the phagosomal compartment. The molecular mechanisms regulating this association-dissociation, which differs with the *iglC* mutant, remain to be experimentally addressed.

### Association of nucleolin with LAMP-1

**Figure 5:**
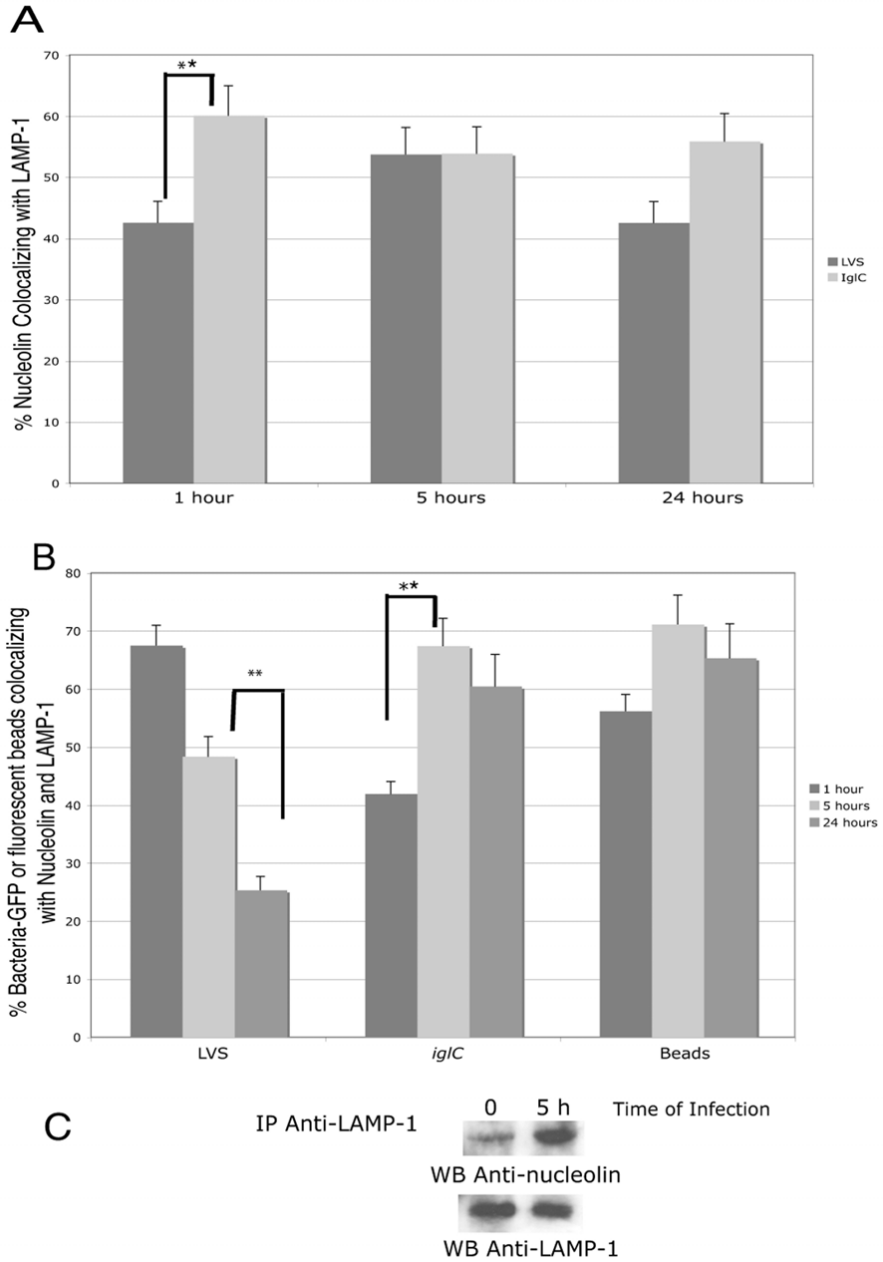
Nucleolin is associated with LAMP-1. Adherent THP-1 cells were infected either by LVS-GFP or *iglC*-GFP and analyzed by confocal analysis, as described in Figure 3. **Part A**: Nucleolin/LAMP-1 co-localization was quantified with Metamorph software. **Part B**: Co-localization of LVS-GFP, *iglC*-GFP bacteria or fluorescent beads with both nucleolin and LAMP-1 was quantified with both Image J and Metamorph softwares. **Parts A and B** result from analysis of six different fields for each time of infection, in five independent experiments. Data are expressed as the mean percentage of co-localization ± SD. * p values <0.01 and ** p values <0.001 (two-tailed unpaired Student's *t* test). **Part C**: 2 mg of proteins solubilized either from cells infected for 5 h with LVS or from uninfected cells (0 h) were immunoprecipitated with anti-LAMP-1 Ab bound to agarose beads. Proteins bound to beads were eluted and analyzed by SDS-PAGE. Immunoblotting was performed either with anti-nucleolin (upper gel) or anti-LAMP-1 MoAbs (lower gel).

We analyzed by confocal microscopy the co-localization of nucleolin with LAMP-1 in adherent THP-1-cells infected either by LVS-GFP or by *iglC*-GFP. Expression of each protein was quantified according to its area, using the Image J software and their % of co-localization was determined using the “co-localization module” of the Metamorph software (**Fig. 5A**). The percentage of nucleolin co-localizing with LAMP-1 was almost constant, from 1 h to 24 h post-infection, with an average value at 45% and at 55% after infection with LVS and the *iglC* mutant, respectively. The constant association of nucleolin with LAMP-1 from 1 h to 24 h suggests that this interaction may occur as well with intact as with already disrupted phagosomes. Transmission electron microscopy (**See Text S1 and Fig. S3**) allowed us to visualize phagosomes with membranes still intact at 5 h (**Fig. S3B**), as at 1 h (**Fig. S3A**), while they were all degraded at 24 h (**Fig. S4C**). These results also indicate that nucleolin/LAMP-1 co-localization occurs in the cells at each stage of infection. In addition, the fact that this percentage is identical, for both LVS and *iglC* mutant strains at 24 h, suggest that nucleolin/LAMP-1 association may occur in different subcellular compartment where LAMP-1 is present [23].

We also analyzed by confocal microscopy the co-localization of GFP-expressing bacteria with both nucleolin and LAMP-1 (**Fig. 5B**). LVS co-localization with both proteins was found to decrease with time of infection (from 65% to 25%). *iglC* co-localization with both proteins reached its maximum at 5 h, with no more variation until 24 h. Fluorescent beads were used as a control of inert particles. Their co-localization with both nucleolin and LAMP-1 presented no significant variation along the 24 h study. The physical interaction between nucleolin and LAMP-1 was confirmed by immunoprecipitation studies (**Fig. 5C**). Proteins solubilized from THP-1 cells infected by LVS for 5 h were absorbed on anti-LAMP-1 Ab immobilized on agarose-beads. A high level of nucleolin associated with LAMP-1 was detected at 5 h. In control, the level of LAMP-1 recovered on anti-LAMP-1 Ab was the same in both samples. These results confirm the nucleolin/LAMP-1 co-localization data observed by confocal microscopy and strongly suggest a physical interaction between the two proteins. Proteins solubilized from non-infected THP-1 cells and treated in the same conditions were taken as control (time = 0). The low level of nucleolin, detected in non-infected cells suggested that interaction of nucleolin with LAMP-1 could occur, even in absence of infection by LVS. This was also suggested by the results obtained with inert particles. However, the level of nucleolin association with LAMP-1 seems to depend on the capacity of the live or inert particles to egress from the phagosomes.

Altogether, these data reveal an interaction between LAMP-1 and nucleolin that had not been previously described. Of interest, it has been shown that the expression of a nucleolin-related protein was moderately up-regulated following phagocytosis of *Mycobaterium paratuberculosis* [24], suggesting that nucleolin could be involved in phagocytosis. One remarkable characteristic of nucleolin is that it shuttles constantly between the nucleus and the cytoplasm [25] and additionally serves in some cell types as a cell surface receptor [26]. Nucleolin has been also described to be present in the endoplasmic reticulum [27]. Thus, nucleolin could play a role in the dynamic network of membrane compartments [28].

In conclusion, we had previously demonstrated that nucleolin, when localized on the cell surface of human monocytes, was involved in LVS infection by its ability to interact with the surface-exposed elongation factor Tu of LVS [13]. We herein confirm the importance of nucleolin expression for LVS binding and its specificity as nucleolin is not involved in binding of another intracellular pathogen as *L. monocytogenes* or an inert particle. We also demonstrate that association of nucleolin with *F. tularensis* during infection continues intracellularly after endocytosis of the bacteria. The co-localization of nucleolin, with bacteria and LAMP-1, in the phagosomes suggests that nucleolin shuttles with *F. tularensis* during the early stages of infection. The dissociation of nucleolin from LVS seems to parallel bacterial release from the phagosomes, as *iglC* bacteria and inert particles remain associated with nucleolin. Recent studies have identified some mammalian host factors required for modulation of phagosome biogenesis and intracellular proliferation of *F. tularensis* within the cytosol [29]. It remains to be seen whether nucleolin plays an active role at the latter stages of *F. tularensis* infection.

## Materials and Methods

### Human cells, bacteria, antibodies and human serum

The human monocyte-like cell line THP-1 (ATCC® Number: TIB-202™) was grown in suspension in RPMI containing 10% fetal calf serum at 37°C in a CO_2_ incubator. Cell viability was measured by Trypan Blue exclusion test. *F. tularensis* Live Vaccine Strain (LVS) and its *iglC* mutant derivative (*iglC*), were kindly supplied by A. Sjöstedt [20], [30] and grown either in Schaedler broth containing vitamin K3 or on chocolate agar plates containing polyvitex (Biomerieux) at 37°C. LVS-GFP and *iglC*-GFP were obtained by transformation of bacteria with pFNLTP6 pgro-gfp, provided by Dr. Zahrt [31]. We used the reference strain of *L. monocytogenes* EGD-e (EGD) (laboratory collection) [32], which was grown in BHI rich medium. EGD-e harbouring the GFP-plasmid (with a GFP encoded by the expression vector pNF8 [33] was grown in presence of 5 µg ml^−1^ erythromycin. Carboxylate-modified microspheres used were 0.5 µm yellow-green fluospheres (Molecular Probes). Polyclonal antibodies (Abs) used were: anti-LVS (Becton Dickinson), anti-nucleolin, anti-calnexin, anti-actin, anti-histone H1 and anti-LAMP1 (Abcam); monoclonal antibodies (MoAbs) were anti-PDI (Abcam) and anti-nucleolin (clone D3) [34]. Secondary antibodies were goat anti-rabbit (GAR) or goat anti-mouse (GAM) conjugated to HRP (Dako) for immunoblotting experiments or to Alexa Fluor cytochromes (Molecular probes) or Cy5-conjugated (Millipore) for confocal microscopy experiments. Human serum AB (PAA) was handled in a manner to preserve complement activity [35].

### siRNA transfection

siGenome SMART pool siRNA targeting nucleolin or histone genes and negative scrambled siRNA control were obtained from Dharmacon. THP-1 cells used in suspension at 10^6^ cells/ml were transfected with 100 nM siRNA, using nucleofactor kit from Amaxa with high efficiency protocol specific for THP-1 cells. After transfection, THP-1 cells were disposed on glass coverslips, at 37°C in presence of 5% CO_2_. After 24 h, phorbol myristate actetate (PMA) was added at 100 ng/ml. After 72 h, LVS-GFP, *iglC*-GFP, *L. monocytogenes*-GFP or fluorescent beads were opsonized for 30 min at 37°C and added on THP-1 transfected cells for 1 h at 37°C. After washing with RPMI containing 10 µg/ml gentamycin to remove extracellular bacteria, the cells were fixed with 4% PFA. Binding of GFP-bacteria and fluorescent beads was analyzed by confocal microscopy and quantified as described below.

### Preparation of solubilized proteins from THP-1 cells

Proteins were solubilized from THP-1 cells by adding to the pelleted cells RIPA lysis buffer containing protease (Roche diagnostics) and phosphatase (Santa Cruz) inhibitors. Proteins were then recovered from the supernatants obtained after centrifugation at 12,000 rpm at 4°C. Protein concentration of the different samples was determined by the BCA assay (Pierce).

### Immunoprecipitation assays

Immunoprecipitation assays were performed by first incubating 30 µl protein G plus/protein A agarose beads (Calbiochem) with anti-LAMP-1 antibodies for 4 h at 4°C. After extensive washes, 2 mg proteins solubilized from THP-1 cells not infected, or infected by LVS for 5 h were added for 16 h at 4°C. After several washes, immune complexes were recovered by heating the samples at 95°C for 10 min in buffer containing SDS.

### Immunoblotting assays

Proteins solubilized from transfected cells or obtained after immunoprecipitation assays were separated by SDS-PAGE and electrotransferred on nitrocellulose membranes (Schleicher & Schuell), using a semi-dry Transblot apparatus (Bio-Rad). Membranes were incubated in Phosphate-buffered saline (PBS) containing 0.05% Tween-20 (PBS-T) and 5% powdered milk, then with the indicated Abs. After extensive washing, HRP-linked secondary Abs were added. Nitrocellulose sheets were washed and binding of second Abs was detected using the ECL Plus kit (Amersham).

### Confocal experiments

Confocal microscopy was performed at the Cell Imaging Facility (Faculté de Médecine Necker Enfants-Malades). Human monocyte-like THP-1 cells were seeded at 1×10^6^ cells/ml on glass coverslips in 12-well bottom flat plates, with addition of PMA. After 48 hours, cells were washed with RPMI and incubated with RPMI-FCS. Cells were then infected for 1 h either with LVS-GFP, *iglC-*GFP, *L. monocytogenes*-GFP or incubated with fluorescent beads, that had been opsonized for 30 min at 37°C in RPMI containing 10% human serum AB (PAA). Cells were then suspended in RPMI containing gentamicin, further incubated for indicated times at 37°C, and treated as previously described [18]. Briefly, adherent THP-1 cells were washed with KHM buffer (110 mM potassium acetate/20 mM Hepes/2 mM MgCl_2_, pH 7.3). Plasma membranes were selectively permeabilized by incubating cells in 50 µg/ml digitonin in KHM for 1 min at room temperature (RT). Cells were then incubated at 37°C with the different primary antibodies followed by incubation for 15 min at 37°C with second antibodies. Cells were then fixed with 4% PFA for 15 min at RT and incubated for 10 min at RT with 50 mM NH_4_Cl to quench residual aldehydes. Cells were washed with PBS and incubated for 30 min at RT in primary antibodies in PBS containing 5% goat serum and 0.1% saponin, which permeabilizes all host cell membranes. After washing, cells were incubated for 60 min at RT with second Abs diluted 1/100 (Alexa Fluor 488 or 546 -labeled GAM or GAR and Cy-5 conjugated GAM or GAR) in the dark. DAPI diluted 1/10,000 was added for 1 min. After washing, the glass coverslips were mounted in Mowiol. Cells were visualized at the appropriate wavelengths under a Leica TSP SP5 confocal microscope with diode (405 nm), argon (458/488 nm), DPSS (561 nm) and helium neon (633 nm) lasers and LASAF program argon (458/488 nm) and helium neon (543 nm) lasers. Quantification of cells and GFP-bacteria or fluorescent beads bound on human cells was performed with Image J software (http://rsb.info.nih.gov/ij) and mean number was calculated on more than 500 cells. Co-localization was first confirmed with Pearson correlation coefficient [36], [37] and Van Steensel coefficient [38] using the JACoP plugin (Just Another Co-localization Plugin) [39]. Then, co-localization of bacteria with nucleolin and/or LAMP-1 was quantified using the “Co-localization” module of Metamorph software.

### Statistical analyses

All confocal experiments have been performed at least five times. A p value <0.01, calculated using the Student's two-tail unpaired *t*-test, was considered statistically different.

## Supporting Information

Text S1 Supporting Materials and Methods. Transmission electron microscopy THP-1 cells were fixed by incubation for 1 h at RT with 2.5% glutaraldehyde in 0.1 M cacodylate buffer (pH 7.2) and washed three times, for 5 min each, in 0.1 M cacodylate buffer. They were then post-fixed by incubation for 1 h at RT with 1% osmium tetroxide in 0.1 M cacodylate buffer. Cells were treated with 1% uranyl acetate for 1 h at RT. Samples were dehydrated in a graded series of acetone solutions and embedded in Epon. Thin sections were cut and stained with 2% uranyl acetate and lead citrate. Sections were observed in a JEOL 1200 EX transmission electron microscope operating at 80 kV and images were recorded with a CCD camera (Megaview, Eloise Ltd).
(0.03 MB DOC)

Figure S1 Adherent THP-1 cells were washed with KHM buffer. Plasma membranes were selectively permeabilized in digitonin. Cells were incubated with anti-calnexin Ab directed against the cytoplasmic tail of calnexin (Part A), followed by incubation with Cy-5 conjugated GAR (blue) or with anti-PDI MoAb directed against the luminal ER protein PDI (Part B), followed by incubation with Alexa 546 GAM (red). Cells were fixed and further incubated with anti-PDI Ab and Alexa 546 GAM (red), both in saponin (Part C). DAPI (white) was added. Cells were visualized with a confocal microscope. We present the photographs of cells with typical morphology and staining.
(6.45 MB TIF)

Figure S2 Adherent PMA THP-1 were infected for 1 h either with LVS-GFP (part A) or iglC-GFP (part B) (green). After washing with gentamycin, the cells were further incubated for 24 h at 37°C. Cells were permeabilized in digitonin and fixed. Cells were then incubated with anti-LAMP-1 Ab (red) and anti-nucleolin MoAb (blue) both in saponin. Cells were then incubated with Alexa Fluor 546-labeled GAR (red) and Cy-5 conjugated GAM (blue) both in saponin. DAPI (white) was added. Cells were visualized with a confocal microscope. We present the photographs of cells with typical morphology and staining. Green arrow points to LVS-GFP bacteria in cytoplasm, yellow arrow points to iglC-GFP bacteria co-localized with LAMP-1.
(6.47 MB TIF)

Figure S3 Transmission electron micrographs of THP-1 monocyte-like cells infected with F. tularensis LVS at 1 h (A), 5 h (B) and 24 h (C). Scale bars are: A, 1 μm, B, 500 nm and C, 5 μm.
(6.22 MB TIF)
