# Supplementary material for: Nucleolin, a Shuttle Protein Promoting Infection of Human Monocytes by Francisella tularensis
Source: PLoS One. 2010 Dec 1;5(12):e14193. doi: 10.1371/journal.pone.0014193 (PMC2995743; doi:10.1371/journal.pone.0014193)
Supplement: Text S1 — Supporting Materials and Methods. Transmission electron microscopy THP-1 cells were fixed by incubation for 1 h at RT with 2.5% glutaraldehyde in 0.1 M cacodylate buffer (pH 7.2) and washed three times, for 5 min each, in 0.1 M cacodylate buffer. They were then post-fixed by incubation for 1 h at RT with 1% osmium tetroxide in 0.1 M cacodylate buffer. Cells were treated with 1% uranyl acetate for 1 h at RT. Samples were dehydrated in a graded series of acetone solutions and embedded in Epon. Thin sections were cut and stained with 2% uranyl acetate and lead citrate. Sections were observed in a JEOL 1200 EX transmission electron microscope operating at 80 kV and images were recorded with a CCD camera (Megaview, Eloise Ltd). (0.03 MB DOC) [file pone.0014193.s001.doc]

**Supporting Materials and Methods**

**Transmission electron microscopy** THP-1 cells were fixed by incubation for 1 h at RT with 2.5% glutaraldehyde in 0.1 M cacodylate buffer (pH 7.2) and washed three times, for 5 min each, in 0.1 M cacodylate buffer. They were then post-fixed by incubation for 1 h at RT with 1% osmium tetroxide in 0.1 M cacodylate buffer. Cells were treated with 1% uranyl acetate for 1 h at RT. Samples were dehydrated in a graded series of acetone solutions and embedded in Epon. Thin sections were cut and stained with 2% uranyl acetate and lead citrate. Sections were observed in a JEOL 1200 EX transmission electron microscope operating at 80 kV and images were recorded with a CCD camera (Megaview, Eloise Ltd).
