# Supplementary figures and images for: Nucleolin, a Shuttle Protein Promoting Infection of Human Monocytes by Francisella tularensis
Source: PLoS One. 2010 Dec 1;5(12):e14193. doi: 10.1371/journal.pone.0014193 (PMC2995743; doi:10.1371/journal.pone.0014193)

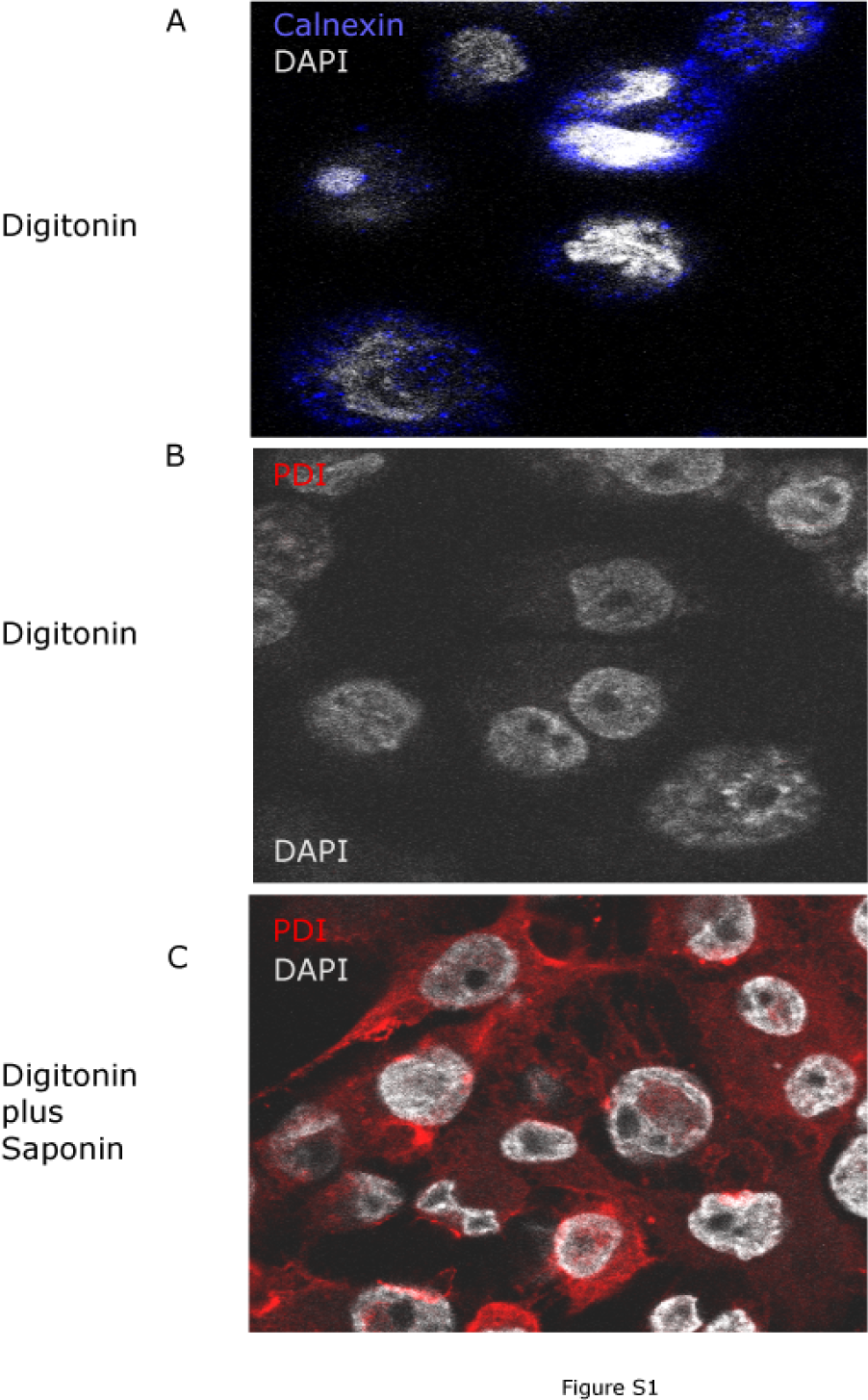

Supplement: Figure S1 — Adherent THP-1 cells were washed with KHM buffer. Plasma membranes were selectively permeabilized in digitonin. Cells were incubated with anti-calnexin Ab directed against the cytoplasmic tail of calnexin (Part A), followed by incubation with Cy-5 conjugated GAR (blue) or with anti-PDI MoAb directed against the luminal ER protein PDI (Part B), followed by incubation with Alexa 546 GAM (red). Cells were fixed and further incubated with anti-PDI Ab and Alexa 546 GAM (red), both in saponin (Part C). DAPI (white) was added. Cells were visualized with a confocal microscope. We present the photographs of cells with typical morphology and staining. (6.45 MB TIF) [file pone.0014193.s002.tif]

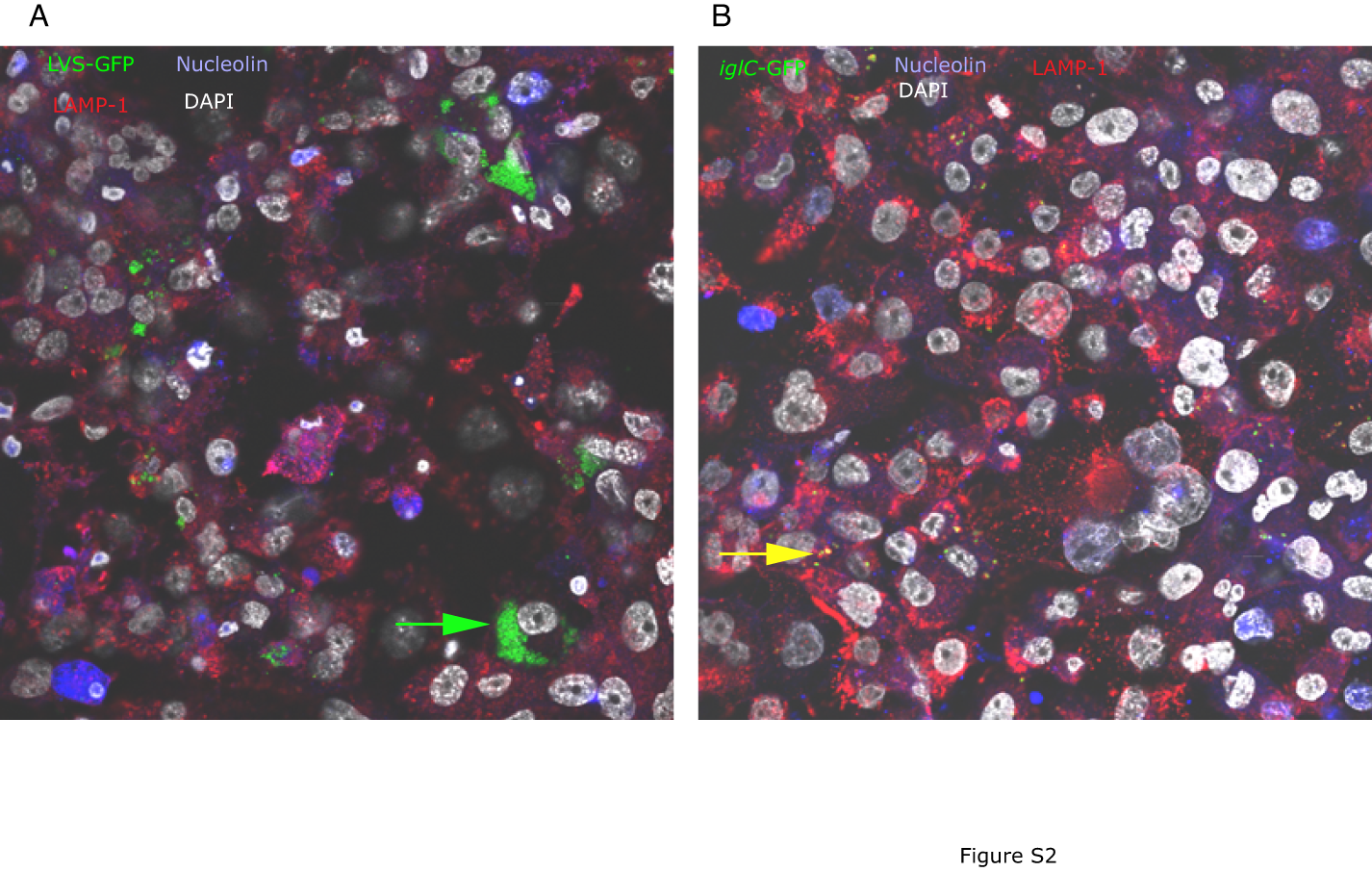

Supplement: Figure S2 — Adherent PMA THP-1 were infected for 1 h either with LVS-GFP (part A) or iglC-GFP (part B) (green). After washing with gentamycin, the cells were further incubated for 24 h at 37°C. Cells were permeabilized in digitonin and fixed. Cells were then incubated with anti-LAMP-1 Ab (red) and anti-nucleolin MoAb (blue) both in saponin. Cells were then incubated with Alexa Fluor 546-labeled GAR (red) and Cy-5 conjugated GAM (blue) both in saponin. DAPI (white) was added. Cells were visualized with a confocal microscope. We present the photographs of cells with typical morphology and staining. Green arrow points to LVS-GFP bacteria in cytoplasm, yellow arrow points to iglC-GFP bacteria co-localized with LAMP-1. (6.47 MB TIF) [file pone.0014193.s003.tif]

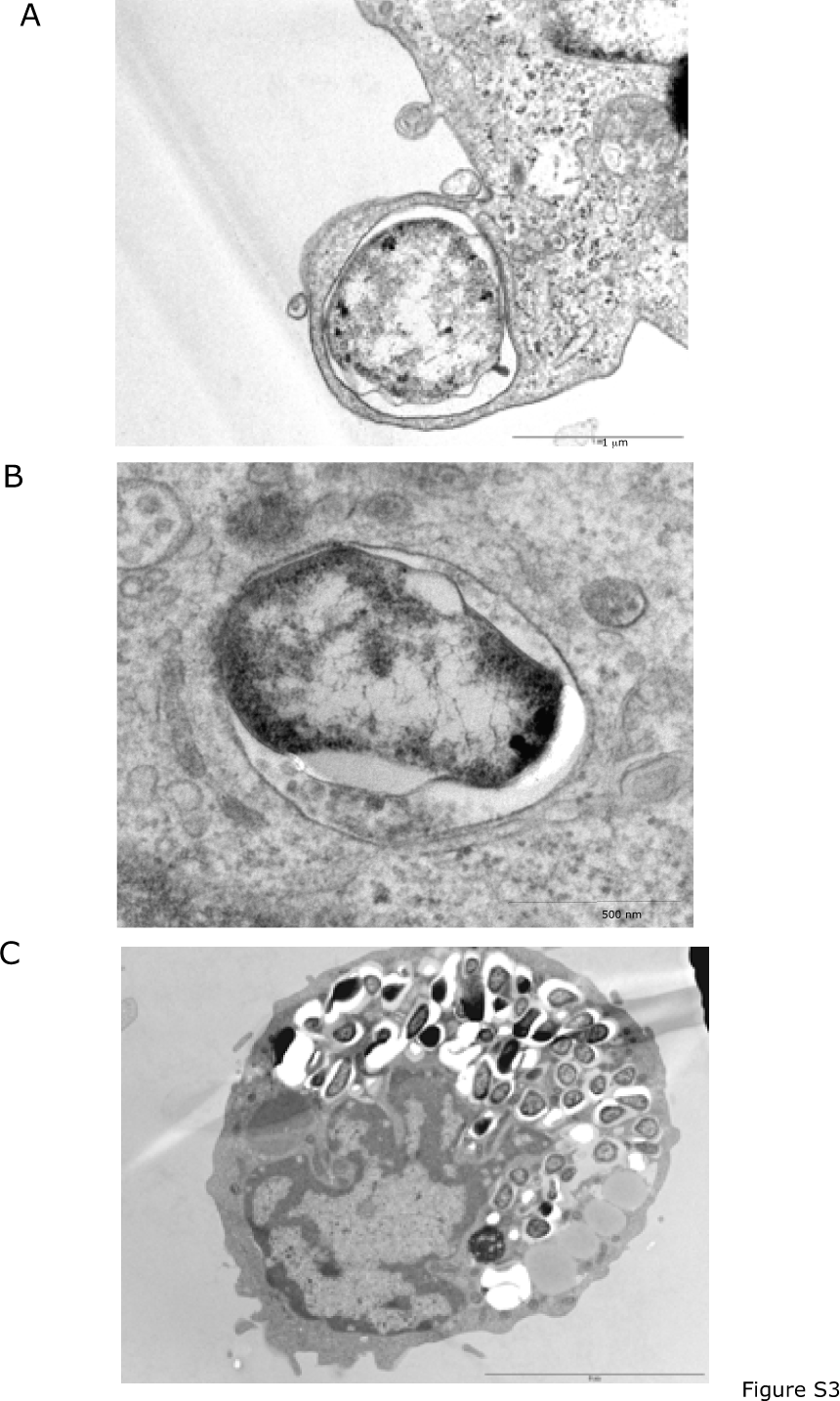

Supplement: Figure S3 — Transmission electron micrographs of THP-1 monocyte-like cells infected with F. tularensis LVS at 1 h (A), 5 h (B) and 24 h (C). Scale bars are: A, 1 μm, B, 500 nm and C, 5 μm. (6.22 MB TIF) [file pone.0014193.s004.tif]
